# Circadian entrainment to red-light *Zeitgebers* and action spectrum for entrainment in the jewel wasp *Nasonia vitripennis*

**DOI:** 10.1007/s00359-023-01672-4

**Published:** 2023-09-22

**Authors:** Yifan Wang, Lijing Jin, Gregor Belušič, Leo W. Beukeboom, Bregje Wertheim, Roelof A. Hut

**Affiliations:** 1 Groningen Institute for Evolutionary Life Sciences, University of Groningen, 9712 CP Groningen, the Netherlands; 2 Department of Biology, Biotechnical Faculty, University of Ljubljana, 1000 Ljubljana, Slovenia

**Keywords:** Circadian rhythm, Red photoreceptor, Action spectrum, Light, Entrainment

## Abstract

Light is the most important environmental cue for the circadian system of most organisms to stay synchronized to daily environmental changes. Like many other insects, the wasp *Nasonia vitripennis* has trichromatic compound eye-based colour vision and is sensitive to the light spectrum ranging from UV to green. We recently described a red-sensitive, ocelli-based photoreceptor, but its contribution to circadian entrainment remains unclear. In this study, we investigated the possibility of *Nasonia* circadian light entrainment under long-wavelength red LED light–dark cycles and characterized the strength of red light as a potential *Zeitgeber*. Additionally, we measured the possibility of entrainment under various light intensities (from 5·10^12^ to 4·10^15^ photons·cm^−2^·s^−1^) and a broader range of wavelengths (455—656 nm) to construct corresponding action spectra for characterizing all circadian photoreceptors involved in photic entrainment. We also conducted electroretinogram (ERG) recordings for each wavelength in the compound eyes. Our findings demonstrate that *Nasonia* can entrain under red light dark cycles, and the sensory pathway underlying the red-light *Zeitgeber* response may reside in the ocelli. Combined with findings from previous research, we pose that blue- and green-sensitive rhodopsin photoreceptor cells function as the major circadian photoreceptors in both circadian entrainment by light–dark cycles and circadian phase shifts by light pulses, whereas the red-sensitive photoreceptor cell requires higher light intensity for its role in circadian entrainment by light–dark cycles.

**Supplementary Information:**

The online version contains supplementary material available at 10.1007/s00359-023-01672-4.

## Introduction

Light is one of the most important external stimuli to entrain behavioural circadian rhythms on a daily basis. The sensitivity and response of animals to light can vary depending on the light conditions, such as wavelength, light intensity, and time of day. In *Drosophila*, multiple photoreceptors play a role in circadian entrainment (Kistenpfennig et al. 2012; Stanewsky et al. 1998; Yoshii et al. 2015). They vary in sensitivity for detecting different levels of light intensities, wavelengths, and duration of the light stimuli (Helfrich-Förster 2020). However, these characteristics of circadian photoreceptors, as well as the mechanisms of circadian regulation, are less well understood in hymenopteran insects. By characterizing a circadian behavioural response to different wavelengths and light intensities, we can assess circadian photoreceptor sensitivity involved in mediating this behaviour. Such characterization will allow for the construction of a circadian action spectrum to define the relative effectiveness of light at different wavelengths for eliciting a circadian response, and to identify the photopigment(s) involved in that response.

As hymenopteran insects possess light-insensitive cryptochrome (CRY), it is thought that visual photoreceptors in the photoreceptive organs are the primary candidates as circadian photoreceptors. Four rhodopsin class photoreceptors have been identified in the jewel wasp *Nasonia vitripennis*, with peak sensitivities at 340 nm (ultraviolet), 450 nm (blue), 530 nm (green), and possibly an additional ocellar photoreceptor at 560–580 nm (red) (Wang et al. 2023). Earlier, we constructed the circadian action spectrum of light for resetting the circadian rhythms (circadian phase shift by light pulses) in *Nasonia,* using monochromatic filters to generate light spectra ranging from 390 to 650 nm. This showed two major peaks for eliciting circadian phase shift by light pulses in the blue (470 nm) and green (540 nm) wavelength range, as well as a smaller peak in the red (620 nm) wavelength range (Wang et al. 2023). The main photoreceptors responsible for the circadian phase shift by light pulses in *Nasonia* appeared to be the blue and green rhodopsin classes (Wang et al. 2023). The finding of an additional circadian phase shift triggered by red light pulses (> 590 nm, (Wang et al. 2023)) was unexpected and it strengthens the hypothesis that *Nasonia* has a spectral sensitivity which extends into red range of the spectrum (Hanai et al. 2008, Lees 1981, Nelson et al. 2009, Saunders 1975). However, the regulation behind red-light pulses-induced circadian response is less well understood.

A common assumption is that insects, particularly bees and wasps, cannot see red light. This assumption is largely based on the fact that the majority of insects belonging to the order of Hymenoptera have three conserved photoreceptor genes in their colour vision system, namely, UV opsin, blue opsin, and green long-wavelength opsin, serving photoreceptors with peak sensitivities around 340 nm, 440 nm, and 530 nm, respectively (van der Kooi et al. 2021). In fact, the visual wavelength range of insects with this trichromatic photoreceptor composition can actually span from UV to red wavelength range (300–650 nm), such as in the honeybee (*Apis mellifera*) and bumblebee (*Bombus terrestris*) (Dyer et al. 2011). Additionally, there are substantial species differences in the visual wavelength range of Hymenoptera and these ranges may include even far-red light. For instance, the solitary bee (*Callonychium petunia*) and sawfly (*Tenthredo campestris*) have photoreceptors with maximal sensitivity around 600 nm (Peitsch et al. 1992). These examples indicate that extreme long-wavelength photoreceptors exist in Hymenoptera that may enable them to see red and far-red light. The small peak at 620 nm in the action spectrum of circadian phase shift by light pulse in *Nasonia* (Wang et al. 2023) suggests that this may also apply for *Nasonia*. Genome annotations and deep transcriptomic analysis demonstrated that *Nasonia* possesses a fourth opsin gene (Davies and Tauber 2016, Werren et al. 2010), which could potentially be red sensitive and explain the circadian responses to red light we previously described (Wang et al. 2023). In addition, electroretinography also revealed different spectral sensitivities in ocelli and compound eyes, with extended long-wavelength sensitivity in the ocelli with an additional peak at around 560–580 nm (Wang et al. 2023). We proposed that this additional ocellar long-wavelength photoreceptor mediated the red-light pulse-induced circadian phase shift (Wang et al. 2023).

In terms of the effect of red light on the circadian system, *Drosophila* can be entrained by red light and such entrainment is mediated by the genes *Rh1* (*Rhodopsin 1*, peak wavelength 480 nm) and *Rh6* (505nm) (Hanai et al. 2008, Helfrich-Förster et al. 2002). Such entrainment is achieved because of a broadening of relative absorption spectra into the red due to overlap with other rhodopsin types (Sharkey et al. 2020). In both humans and rats, red light beyond the sensitivity range of rods can entrain their circadian physiology and behaviours, as well as cause phase shifts (Dauchy et al. 2015; Hanifin et al. 2006; McCormack and Sontag 1980). Therefore, it is worth further investigating the light entrainment photoreceptors of *Nasonia*, in particular whether *Nasonia* can be entrained under red light, and whether the red-absorbing visual pigment plays a role in photoentrainment. Additionally, various circadian behaviours may be regulated by different photoreceptors and through different molecular pathways. In *Drosophila*, rhodopsin-expressing photoreceptor cells in the compound eyes as well as the brain photoreceptor cryptochrome (CRY) contribute to light entrainment and circadian phase shifts, but through different mechanisms, and the photoreceptors in the eyes contribute less to phase shift regulation than the brain photoreceptor CRY (Kistenpfennig et al. 2012; Senthilan et al. 2019; Stanewsky et al. 1998; Yoshii et al. 2015).

For a better understanding of the various pathways of circadian photoreception in *Nasonia*, we will assess the spectral sensitivity of the circadian photoreceptors mediating light entrainment and examine whether different photoreceptors are involved in light entrainment by light–dark cycles or phase shift by light pulses in *Nasonia*. For this, we entrained *Nasonia* under long-wavelength red lights and characterized the strength of red light as a potential *Zeitgeber*. We established an action spectrum for light entrainment by light–dark cycles to compare the circadian spectral sensitivity for phase shift by light pulses.

## Materials and methods

### Experimental lines and maintenance

The standard laboratory strain of *Nasonia vitripennis*: AsymCx was used in all experiments. This is a wild-type strain that has been kept in the laboratory since 1971 and was cured of the *Wolbachia* infection with antibiotics (Perrot-Minnot and Werren 1999, van den Assem et al. 1980). Its whole genome is sequenced and annotated (Werren et al. 2010). The wasps were kept in plastic vials (70 × 20 mm) with approximately 20–30 animals per vial to avoid overcrowding, in a temperature- and humidity-controlled incubator (20 ± 1 °C, 50–55% RH). They were maintained under a light–dark cycle of 18:6 LD (60 lum/sqf) to prevent diapause induction. Pupae of *Calliphora *spp*.* flies were provided as hosts, and wasps were rehosted on a 21-day cycle with about 15–20 *Nasonia* females on 30–50 fly hosts each generation.

To produce wasps for experimental use, 1–2 days old, mated females were individually separated into small cotton-plugged polystyrene tubes (60 × 10 mm) and supplied with three to four *Calliphora *spp*.* pupae; the emerging offspring was used in the experiments. This method was used to avoid overcrowding and to achieve maximal survival and life span of individual wasps during experiments.

### Locomotor activity measurements

For all locomotor activity measurements, unmated females were used, because mated females have reduced rhythmicity and life span (Paolucci et al. 2019). To ensure females were unmated, they were sexed and separated in the black pupal stage (approximately, 17–18 days after eggs were laid). Black pupae were put into cotton-plugged polystyrene tubes with a piece of filter paper dipped in sugar water in single-sex groups of 10–15 pupae per tube. Once eclosed, 1–2 days old unmated females were transferred into locomotor activity tubes (6.5 × 0.5 cm), of which one-quarter was filled with agar food (30% sucrose, 1.5% agar, 0.1% nipagin); the activity tubes were closed with black plastic plugs at both ends. Locomotor activity tubes (*n* = 32 per experiment) were loaded into the Drosophila Activity Monitoring System (DAMS, TriKinetics, Waltham, USA) to record locomotor activities. Locomotor activity in the DAM monitor was detected by infrared light beam breaks through the middle of the tube and stored every minute. The DAM monitors were installed into light-tight boxes (23 × 14 × 32 cm), in a temperature- and humidity-controlled climate room (18 ± 1 °C, 50–55% RH). Within each light-tight box, an LED light source was installed and at least one diffuser sheet was placed under the LED light and above the animals to ensure all animals received an equal amount of light.

### Light entrainment experiments

In total, nine commercially available LED light sources (Intelligent LED solutions, Berkshire, UK) were used in the light entrainment experiments (Table 1). Individual LEDs were installed separately in each light-tight box. For red-light entrainment experiments, the three long-wavelength LED lights (590 nm, 625 nm, and 656 nm) were used (Table 1). We also noticed that the precise peak wavelengths of the LED lights deviate slightly from the peak wavelengths reported by the manufacturer (Table 1). These LEDs resulted in illumination at approximately 1.75 × 10^15^, 2.22 × 10^15^, and 3.30 × 10^15^ photons·cm^−2^·s^−1^ intensity, respectively. The range of conditions under which entrainment is achieved can be used to characterize and compare the strength of these three red LED lights as *Zeitgebers*. The range of circadian entrainment to light–dark cycles with different period length (T-cycles) will increase with *Zeitgeber* strength and is defined as the range between the shortest T-cycle and the longest T-cycle in which entrainment is observed. The percentage of entrainment will follow a bell-shaped curve (0–100–0%) under a full T-cycle range. Increasing *Zeitgeber* strength will necessarily cause the increase in the number of entrained individuals under the range of T-cycles. Stronger *Zeitgebers* will also decrease the variation in phase angles of entrainment observed (the amount leading or lagging), and the linear relationship between T-cycle duration and phase angle of entrainment will thus show a flatter slope with increasing *Zeitgeber* strength (Floessner and Hut 2017).

**Table 1:** LED light information for the entrainment experiment

LED light	Colour	Wavelength (nm)		Measured wavelength (nm)	Bandwidth (nm)
ILH-OP01-DEBL-SC221-WIR200	Deep blue	455		438	81
ILH-PO01-BLUE-SC221-WIR200	Blue	470		470	94
ILH-PO01-VEGR-SC221-WIR200	Verde green	505		493	98
ILH-PO01-TRGR-SC221-WIR200	True green	528		523	109
ILH-OP01-PCGR-SC221-WIR200	Phosphor Converted green	566		554	288
ILH-PO01-YELL-SC221-WIR200	yellow	590		618	249
ILH-PO01-RDOR-SC221-WIR200	Red orange	617		623	77
ILH-PO01-RED1-SC221-WIR200	Red	625		631	78
ILH-PO01-HYRE-SC221-WIR200	Hyper red	656		661	89

Wavelength (nm): manufacturer-provided information on the peak wavelength of the LED lights. Measured wavelength (nm): the actual peak wavelength of the LED lights measured with a spectrometer in the laboratory. Bandwidth (nm): spectrometer-measured bandwidth of the LED lights

Our approach was to expose *Nasonia* to short periods (4 h) of red light and to characterize the range of entrainment by challenging their circadian system and reducing the T-cycle away from 24 h (the circadian period *tau* in DD for *Nasonia* is ~ 23–27 h). Animals were entrained with the red-light LED sources under different T-cycles ranging from 19 to 24 h for at least 2 weeks. The length of the light period for each entrainment was kept constant at 4 h and only the length of the dark period for each entrainment varied from 15 to 20 h. To avoid overheating during the 4-h light phase, LED lights were equipped with a large heat sink, continuously cooled with a light-tight forced airflow. During a pilot experiment, a temperature increase of 2–3 °C was noticed during the 4-h light phase. We therefore adjusted the light phase to a "pulse" cycle of 10 min on and 20 min off for 4 h to avoid possible temperature entrainment. Still, there was a 0.3–0.5 °C temperature elevation during the light phase. Programmable timers and controllers were used for controlling the light cycles (ChronTrol electronic programmable timer, ChronTrol cooperation, San Diego, USA). In total, six different T-cycles were tested with the three different light sources and the achieved sample size for each condition was between 12 and 27 wasps.

To entrain *Nasonia* under different wavelengths of LED light, all nine LED lights were tested individually (455 nm, 470 nm, 505 nm, 528 nm, 566 nm, 590 nm, 617 nm, 625 nm, and 656 nm). The entrainment protocol for this was 4 h of light and 17 h of dark (*T* = 21 h) for 3 weeks. To avoid overheating during the 4-h light phase, LED lights were equipped with a large heat sink, continuously cooled with a light-tight forced airflow, and intermittently turned on with a pulse cycle of 10 min on and 20 min off. Various combinations of diffusor sheets and neutral density filters (LEE Neutral Density filters 0.5–1.2 ND) were used to adjust light intensities in each lightbox. In total, nine different wavelengths and five to six different light intensities (4.89 × 10^12^ to 4.04 × 10^15^ photons·cm^−2^·s^−1^) per wavelength were tested (*N* = 13–44 wasps). These dose–response measurements at each wavelength were then used to construct the corresponding action spectra.

### Temperature entrainment experiments

Despite our efforts to reduce heat production by the LED sources, we measured a slight temperature elevation of 0.3–0.5 °C during the 4-h lights-on phase. We therefore performed temperature entrainment experiments as a control to rule out the possible entrainment effect due to these temperature fluctuations. To achieve a very stable temperature environment, a 32-channel DAM monitor was positioned inside an insulated polystyrene box (33 × 36.5 × 32 cm) tightly surrounded by water tubes. Circulating water inside the water tubes was provided by a pumping water bath device (LAUDA Ecoline staredition RE204). To mimic the temperature cycle during the LED light entrainment experiments, the temperature cycle of the water bath was set to 24 h, using 4 h of increased temperature and 20 h of constant temperature. The constant temperature was set to room temperature (18 °C), and the increased temperature was tested at six different temperatures, ranging from 18.5 °C to 23 °C. Animals were tested under those temperature cycles for 3 weeks under total darkness. The insulated polystyrene box was covered by a thick black light-tight photographic dark-room fabric to prevent any light input, and the climate room in which the system was placed was kept in darkness at all times as well.

### Electroretinogram (ERG) recordings

The spectral sensitivities of the photoreceptors in the compound eyes and in the ocelli were determined with extracellular electroretinogram recordings (ERG). The wasps were immobilized using beeswax and resin and pre-oriented on a mini-goniometer with respect to the light stimulus to yield maximal light responses. The goniometer was mounted on a rotatable goniometric and xyz stage that carried a micromanipulator (Sensapex, Oulu, Finland). The electrodes, pulled from borosilicate glass on a horizontal puller (P-2000, Sutter, Novato, CA, USA) with a 1–5 μm tip, were filled with insect saline (0.67% NaCl, 0.015% KCl, 0.012% CaCl_2_, 0.015% NaHCO_3_, pH 7.2) and inserted just below the cornea of the compound eye or into the head capsule next to individual ocelli. Light stimuli were produced with a 75 W xenon arc lamp (Cairn, UK), filtered with a monochromator (B&M Optik, Germany) and a motorized continuously variable neutral density filter (Thorlabs, Germany), or with a custom array of monochrome LEDs ranging from 365 to 700 nm in ~ 15 nm interval (Roithner Laser, Austria), synthesized with a diffraction grating (Thorlabs, Germany). The light sources were adjusted with a radiometrically calibrated flame spectrophotometer (Ocean optics, USA) so that the maximal light intensity in the focal plane of the visual organ was 1.5 × 10^15^ photons·cm^−2^·s^−1^. LED stimulation for ERG was at the same wavelengths as for the behavioural experiments (455 nm, 470 nm, 505 nm, 528 nm, 566 nm, 590 nm, 617 nm, 625 nm, and 656 nm), with an additional LED at 540 nm.

#### Determination of entrainment and statistical analysis

All raw behavioural data were initially examined using Chronoshop (Spoelstra 2018), to inspect the actograms and centre of gravity per day. All behavioural data were further analysed and plotted in R (v. 4.1.2) (R Core Team 2021). Rethomics R framework (Geissmann et al. 2019) was used to analyse DAM monitor recordings and for plotting actograms and calculating periodograms. Moving (if the activity of the animal per minute is bigger than 0) is plotted in all actograms. Because of the extreme light conditions used (small T-cycles and “10 min on and 20 min off” light period), many wasps showed unusual behavioural profiles. Therefore, we applied the following rules to determine entrainment, to avoid over- or underestimation of the possibility of entrainment. First, animals that lived shorter than 10 days were considered too short-lived and were removed from the analysis. Animals that showed long gaps in their activity, such as a week of absent activity, were also removed from the analysis. Secondly, based on previous research, a relatively high percentage of *Nasonia* are naturally arrhythmic (Floessner et al. 2019, Paolucci et al. 2019; Wang et al. 2023). We could not distinguish between naturally arrhythmic wasps and light condition-induced arrhythmic wasps; thus, arrhythmic wasps were excluded from the analysis. For instance, wasps that showed hyperactivity throughout the days were considered arrhythmic. Lastly, periodograms (with R package zeitgeber), average activity profiles, and centre of gravity per day were calculated for each animal in R, and entrainment was judged by combining this information. Individuals were considered entrained when their activity mainly concentrated during the day, the calculated period was close to the given T-cycle, and/or the centre of gravity followed one vertical line approximately. Individuals were considered free running when there was a clear drifting in their activity, the calculated period was not close (> 0.5 h) to the given T-cycle or period could not be determined, and/or there was a clear drifting in the centre of gravity. Animals were considered free running, mainly when the free running pattern covered all days of the T-cycle.

To analyse the effect of wavelength and T-cycle on entrainment, we selected only the rhythmic wasps, where free running wasps were referred to as 0 and entrained wasps were referred to as 1. We applied a binomial generalized linear model (GLM) to take into account the sample size variations for each condition. In the applied GLM model, the binary response (entrained or free running) was defined as the response variable, whereas wavelength, T-cycle, and the interaction between wavelength and T-cycle were defined as predictor variables [glm(binary response ~ wavelength*Tcycle, family = “binomial”)]. We used Chi-square test for GLM model comparison and selection, and we used likelihood ratio F tests (Anova(m1) from the car package in R (Fox and Weisberg 2011)) in the final model. The percentage of entrainment was calculated as the number of entrained animals divided by the total number of rhythmic (entrained and free running) animals for visualization. To determine the average phase angle of entrainment in the T-cycle experiments, the first 4 days of non-entrained activity were removed before calculating the average centre of gravity (phase angle of entrainment) in hours after lights on. The effect of wavelengths and T-cycles on phase angle of entrainment was then analysed using a linear effect model [lm(phase angle of entrainment ~ wavelength + Tcycle)]. Post hoc tests were used to compare the differences between wavelength (i.e. the slopes of the linear regression).

For analysing the circadian action spectrum of entrainment, we firstly applied a similar binomial generalized linear model (GLM) to assess the effect of wavelength and light intensities on entrainment [glm(binary response ~ wavelength + light intensity, family = “binomial”). To compare *Nasonia*’s sensitivity to different wavelengths and construct the circadian action spectrum, we then performed dose–response analysis by fitting a series of sigmoidal curves using R packages drc (Ritz et al. 2015). In the applied drc model, the percentage of entrained animals was defined as response variable, light intensity was defined as predictor variable, and all data were grouped by wavelength. As the proportion of entrainment is between 0 and 1, dose–response curve parameters were estimated by nonlinear regression using a two-parameter (effective dose and steepness of the curves) sigmoidal function. Effective dose (ED) was then derived from the dose–response curves as an indication of *Nasonia*’s spectral sensitivity. Different levels of effective dose were calculated due to the non-univariance of the slopes. By setting the response threshold at 30%, 50%, and 80%, the model could calculate how much light intensity was needed to produce this level of response based on the fitted Hill equation. The same dose–response analysis was performed for the electroretinogram recordings to determine *Nasonia*’s sensitivity to different wavelengths in terms of electroretinogram responses in the compound eye.

## Results

### Red-light entrainment in *Nasonia vitripennis*

We previously identified four photoreceptor classes in *Nasonia* through electroretinogram measurements, with peak wavelengths at 340 nm (UV), 450 nm (blue), 530 nm (green), and 560–580 nm (ocellar red, (Wang et al. 2023)). By entraining *Nasonia* under long-wavelength LED lights (590 nm, 625 nm, and 656 nm), it allowed us to examine the possibility of red-light entrainment in *Nasonia* and to assess which photoreceptor mediates entrainment under long-wavelength range. By solely decreasing the night length and thereby reducing the T-cycles, we could characterize and compare the efficiency of these three wavelengths as *Zeitgeber*. This way, we challenged the circadian system to the limit, as it is harder for animals to entrain under a T-cycle that is significantly different from their intrinsic circadian period (*tau*).

**Fig. 1:**
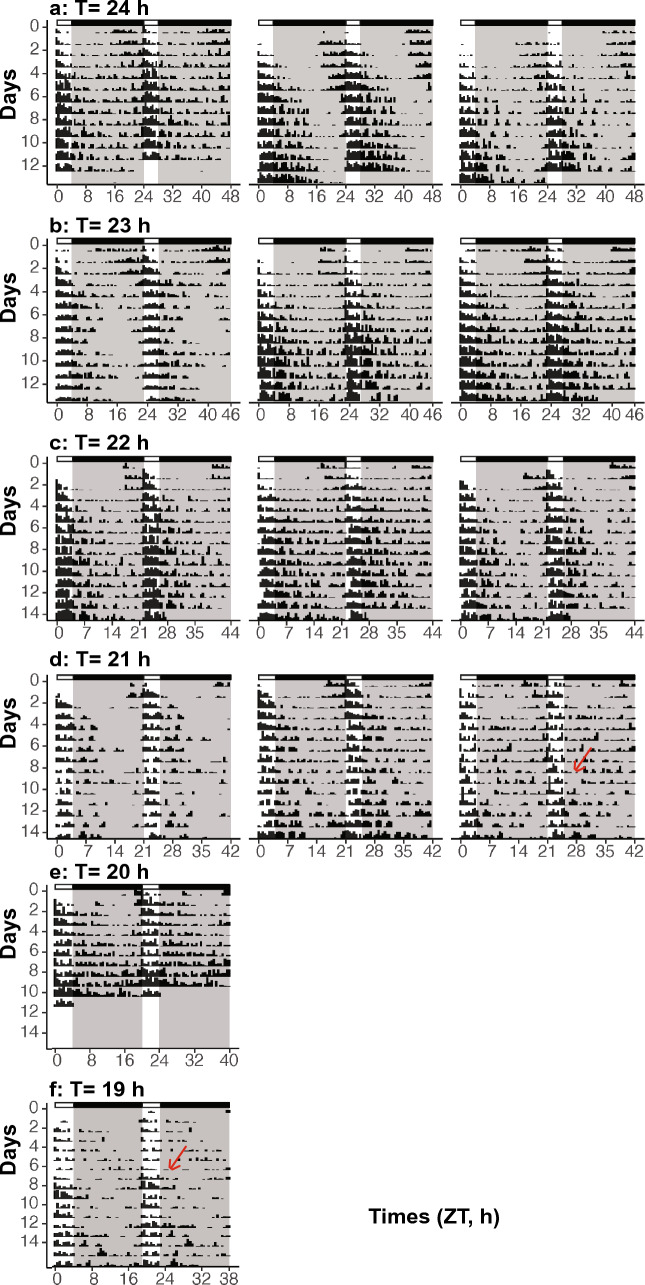
Representative activity patterns of entrained *Nasonia* under different T-cycles under far-red light (656 nm). a-f Three representative actograms in double-plot of *Nasonia* entrained under each T-cycle ranging from a 24 T to f 19 T. T-cycle length is the sum of the light and dark phase lengths. The light phase was kept constant at 4 h and only the dark phase varied in different T-cycle experiments. Light/dark cycle is indicated on top and in the background of each panel, where white is the light phase and black/grey is the dark phase. Red arrows in the figures indicate when relative coordination occurred in the example animals (relative coordination: an animal shows the alternation between free running and entrainment). Time scale starts at ZT0, the start of light on

Arrhythmic wasps were excluded from the analysis to determine phase angles of entrainment. The percentage of arrhythmicity varied from 0 to 28% under the tested conditions (Supplementary Information Tab. s1). The percentage of arrhythmic wasps increased significantly with the shortening of the T-cycle (quasibinomial generalized linear model [glm], T-cycle, *p* < 0.001). Additionally, under each light condition, animals that did not survive for at least 10 days were excluded (6% to 53%) from the analysis to better distinguish between entrainment and free running (Supplementary Information Tab. s1 and Online Resource 1). The remaining individuals either became entrained to the red lights or showed free running behaviour. Multiple analyses were combined to determine entrainment of the wasps (actogram, periodogram, average activity profile, and daily centre of gravity plot). These results showed that our experimental setup was suitable to induce circadian entrainment and that there was entrainment under red light. Surprisingly, even under far-red light (656 nm) and under some of the short T-cycles, some animals were able to entrain (example of entrained animals in Fig. 1, Supplementary Information Fig. s1-s2, Online Resource 1). However, considering the extreme T-cycles and light on–off schemes applied in the experiments, relative coordination occurred in some animals that the animals exhibit the transition between entrainment and free running and some alternations between the two stages (Fig. 1, Supplementary Information Fig. s1-s5, Online Resource 1). To avoid over- and under estimation of entrainment, animals were classified as free running as soon as an indication of a free running circadian component is detected in the analysis. Although some free running animals showed period almost equal to the entrained T-cycles, this could be induced because of direct behavioural responses (light masking) to the light stimulation.

**Fig. 2:**
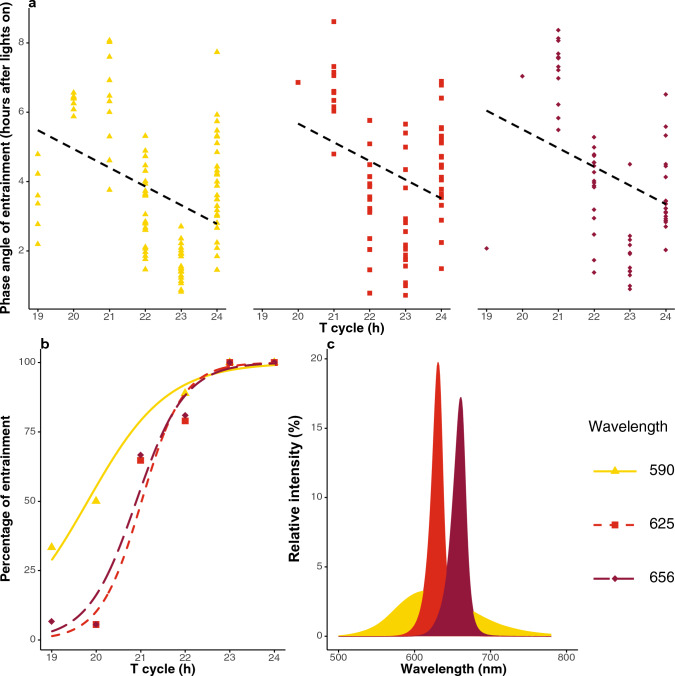
Red-light entrainment of *Nasonia* under three long-wavelength lights (590 nm, 625 nm, and 656 nm). **a** The phase angle of entrainment of *Nasonia* under each wavelength and each T-cycle. The phase angle of entrainment is calculated in hours after lights on. Black dashed lines indicate the model prediction from the linear model and post hoc test was used to compare the steepness of the slopes. **b** The percentage of entrainment of *Nasonia* under each wavelength and each T-cycle, percentage of entrainment is calculated as the number of entrained *Nasonia* divided by the total number of rhythmic animals (entrained and free running). The fitted lines indicate the model prediction from the binominal generalized linear model. **c** Spectra of the 590 nm, 625 nm, and 656 nm LED light sources that were used in this study. Relative intensity is calculated as light intensity divided by the averaged value of each light, respectively. The colours in the graph correspond to the subjectively perceived colour of LED lights used

Considering the broad absorption spectral profiles of the rhodopsins, we purposely selected three commercially available long-wavelength LED lights (590 nm, 625 nm, and 656 nm) that were partially overlapping with the green photoreceptors (590 nm), almost overlapping (625 nm) or completely outside (656 nm) the visual range of the green photoreceptors (Fig. 2c). Under each of the three long-wavelength LED lights, the wasps showed circadian entrainment and responded to shortening of the T-cycle (Fig. 2a). All wasps were fully entrained under all three long-wavelengths LEDs when given a T-cycle of 23 h or 24 h. The number of free running animals increased with the shortening of the T-cycle (example of free running animals in Supplementary Information Figs. s3–s5). As predicted, the percentage of entrainment was significantly affected by T-cycles (binomial generalized linear model [glm], T-cycle, *p* < 0.001) as fewer animals became entrained under shorter photoperiods (Fig. 2b). There was a slight significant interaction between wavelength and T-cycle (*p* = 0.05). The effect of wavelength on the percentage of entrainment was also significant (*p* < 0.001).

Due to the shor life span of *Nasonia*, we could not add DD treatment after entrainment. Instead, we chose to analyse the phase angle of entrainment under each *Zeitgeber* and to compare the strength between the long-wavelength *Zeitgebers*. Although there are still some variations in the phase angle of entrainment, there was a general regression that phase angle of entrainment decreased with the increase of the T-cycles (Fig. 2a). Based on this analysis, the relative effectiveness of the three *Zeitgebers* for eliciting a circadian response showed no significant difference in the range of entrainment between the three wavelengths (linear effect model and post hoc test; Fig. 2a). These combined data showed that *Nasonia* can be entrained to long wavelengths of light, up to at least 656 nm.

When the LED lights were turned on, it caused a small temperature increase of ~ 0.3–0.5 °C. To exclude the possible effects of temperature on entrainment, we did a controlled temperature cycle experiment. We housed the wasps in constant darkness, where we increased the temperature daily by 0.5, 1, 2, 3, 4 or 5 °C for 4 h, after which the temperature returned to baseline for the remaining 20 h. For the two smallest temperature cycles (0.5 and 1 °C), only 1 out of 21 or 25 wasps, respectively, showed entrainment (Supplementary Information Fig. s6, example of entrained animals in Supplementary Information Fig. s7-s9, Tab. s2, Online Resource 2). The proportion of entrained animals increased gradually when the temperature was raised beyond 1 °C and all wasps became temperature entrained with a temperature amplitude of 4 and 5 °C (example of free running animals in Supplementary Information Fig. s10–s12, Table s2). This demonstrated that *Nasonia* can be temperature entrained, but for a cycle where the temperature is increased with a minimum of 1 °C under 24 T. This indicates that the entrainment experiment under LED lights and shorter T-cycles with a temperature cycle of ~ 0.3–0.5 °C was not mediated by the temperature, but by light.

### Circadian action spectra for entrainment in *Nasonia vitripennis*

**Fig. 3:**
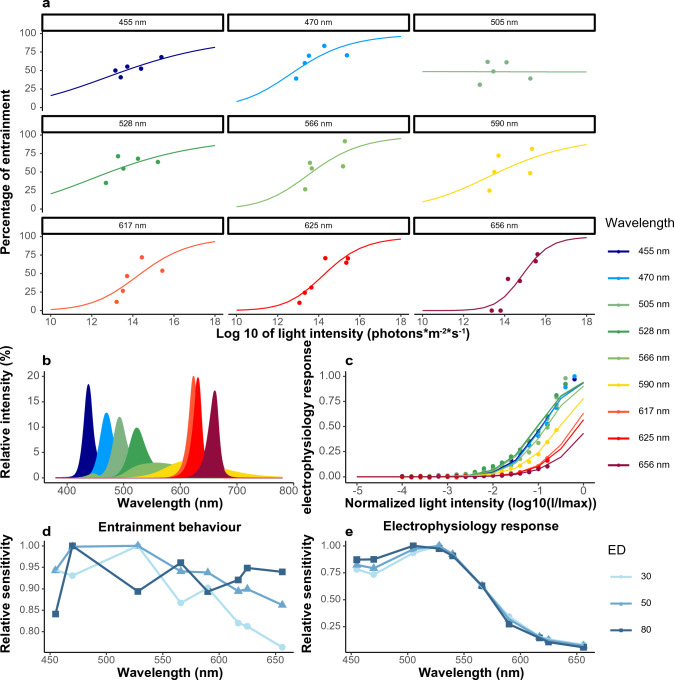
Dose–response curves for light entrainment and corresponding action spectra. Nine different LED lights with wavelengths from 455 to 656 nm were tested. **a** Dose–response curves of the percentage of entrained animals under nine different wavelengths and five to six different light intensities per wavelength. **b** Spectra of all LED lights used in this study was shown. Relative intensity represents the ratio of light intensity value divided by the averaged light intensity value of each light. **c** Dose–response curves of the electroretinogram responses of *Nasonia*’s compound eye to nine different wavelengths. **d** Circadian action spectra of entrainment behaviour were derived from the dose–response curves in a by determining the light intensities that were necessary to entrain 30%, 50%, and 80% of the wasps at each wavelength. The effective dose of 505 nm was excluded from the action spectra, as the dose–response curve was unable to fit in that data. Additional circadian action spectra with corrected wavelengths were also plotted and are supplied in Supplementary Information Fig. s13. **e** The electroretinogram action spectra were derived from the dose–response curves in **c** to compare with the action spectrum of circadian light entrainment (only 9 wavelengths are plotted here, but an extra wavelength 540 nm was included in the final dose–response analysis). The light intensities needed to trigger 30%, 50%, and 80% of the electroretinogram response in *Nasonia*’s compound eye were determined. Relative sensitivity is calculated as 1/effective dose (light intensity) and normalized to the maximum sensitivity. The colours in **a**–**c** correspond to the wavelength of the lights. The colours in d–e indicate ED30 (light blue), ED50 (blue line), and ED80 (dark blue line)

We further investigated the spectral sensitivity of *Nasonia* for circadian photoentrainment. To characterize the photoreceptors involved in light entrainment, we tested the circadian light entrainment under a relatively short T-cycle (21 T), and at different light intensities. We determined the action spectrum of light entrainment using nine commercially available LED lights, with spectra ranging from 455 nm (deep blue) to 656 nm (dark red) (Fig. 3b, Supplementary Information Tab. s3, and Online Resource 3) taking into account the interactive involvement of several photoreceptors (Wang et al. 2023). Additionally, we used electroretinogram recordings in the compound eyes at different light intensities, but with monochromatic lights for the same nine wavelengths and an extra 540 nm (Fig. 3c). Light intensity had a significant effect on the entrainment as the percentage of entrainment increased with increasing light intensity (Fig. 3a, binomial generalized linear model [glm], light intensity, *p* < 0.001). The effect of wavelengths on the entrainment was also significant (binomial generalized linear model [glm], wavelength, *p* < 0.001).

The fitted dose–response curves under each wavelength for both behavioural entrainment and electroretinogram responses, the wavelength spectra of the LED lights, and the corresponding action spectra are presented in Fig. 3. The percentage of entrainment followed the dose-dependent sigmoidal curves under almost all LED lights, except for 505 nm (Fig. 3a). The effective dose parameter in the dose–response curve fitting was significant in all wavelengths (drc model, *p* < 0.05, Tab. s4). Notably, the dose–response curves under different wavelengths had different slopes. The slopes were the highest for the red LEDs, whereas the slopes for the blue and green LEDs were lower. The slope parameter in the curve fitting was only significant for wavelengths 625 nm and 656 nm (*p* < 0.05, Tab. s4). However, the differences in slopes do indicate that the circadian light input deviates from the principle of univariance and thus suggests that more than one photopigment, residing in various photoreceptor cells, is involved in regulating the circadian light entrainment behaviour. Therefore, we derived three action spectra of light entrainment to examine how their spectral sensitivities may change at different levels of light intensity by determining the light intensities needed (or, effective dose) to entrain 30% of the wasps (low light level), 50% (intermediate light level), and 80% (high light level) (Fig. 3d). The electroretinogram responses of the compound eye to these ten specific wavelengths also showed non-univariance in slopes (Fig. 3c). Here, the electroretinogram responses were lowest under far-red lights, indicating that the photoreceptors for red light are not located in the compound eye, where the sensitivity to far-red was very low. Electroretinogram recordings of the ocelli under a full matrix of intensity and wavelength were previously measured (Wang et al. 2023). The three action spectra at low, medium and high light intensity of the electroretinogram responses were also derived to enable comparison to the circadian action spectra (Fig. 3e).

The relative sensitivity of *Nasonia*’s electroretinogram response in the compound eye showed a broad peak ranging from blue to green light (450–530 nm), and the relative sensitivity dropped down gradually to almost zero in the longer-wavelength range (Fig. 3e). Since the peak wavelengths of LED lights slightly deviate from the manufacturer report, we also plotted Fig. 3d with the more precise peak wavelengths (Supplementary Information Fig. s13). Due to the wide bandwidth of the LED lights, we cannot pinpoint the relative peak sensitivity of *Nasonia*’s circadian response. Thus, we focused on comparing the shapes of the action spectra instead. There is almost no difference between the three action spectra of the electroretinogram response for different effective doses ([ED]30, ED50, and ED80, Fig. 3e). On the contrary, the three circadian entrainment action spectra showed major differences (Fig. 3d). When given a low light level (ED30), the sensitivity was high between 450 and 528 nm with a peak sensitivity at ~ 528 nm, and a smaller peak at ~ 590 nm. The peak sensitivity wavelength can only be roughly estimated in this setup due to the limited wavelengths of the commercially available LED lights. The overall spectral sensitivity decreased with increasing wavelength, and there was almost no entrainment at the longest wavelength (656 nm) with low light intensity (< 10^–14^ photons∙m^−2^·s^−1^, Fig. 3a). In comparison, the range of the circadian action spectrum became broader at higher light levels. At the intermediate light level (ED50), the sensitivity was highest between 470 and 528 nm and a relatively strong sensitivity was observed in the longer-wavelength range (> 550 nm). The spectral sensitivity showed more fluctuations with multiple peaks across the spectrum at high light levels (ED80). There were almost reversed patterns between ED30 and ED80 in terms of the peaks and valleys. Although the maximal sensitivity remained in the blue light region, the sensitivity in the longer-wavelength (red light) region was elevated compared to ED30 and ED50. This indicates that red light is a potent *Zeitgeber* for behavioural circadian entrainment, especially at medium to high light intensities. Overall, the relative spectral response in electroretinogram seems unaffected by light intensity (Fig. 3e), whereas the circadian response does not. Thus, the photoreceptors in the ocelli, but not in the compound eyes are likely to be involved in sensing red light.

## Discussion

To confirm the sensitivity of circadian behaviour to red light in the jewel wasp (*Nasonia vitripennis*), we entrained *Nasonia* to three commercially available LED lights with long wavelengths (590, 625, 656 nm) under different light/dark cycles (T19–T24). The locomotor activity of all wasps became synchronized to the red light/dark cycle when the T-cycle was closer to the internal circadian period under DD (23–27 h). To further identify the relative spectral sensitivities and the involvement of multiple circadian photoreceptors for light entrainment, we constructed action spectra for photoentrainment under nine LED lights of various wavelengths (455–656 nm). We combined behavioural data and electroretinogram recordings to construct the action spectra. We found that the maximum sensitivity for entrainment was between the blue and green wavelength range and the sensitivity to red lights increased when light intensity increased. These results suggest the involvement of the blue (450 nm) and green (530 nm) opsin classes as the main photoreceptors for entrainment, as well as an additional photoreceptor for red light that may not be located in the compound eye. The red rhodopsin class (560–580 nm) that we identified earlier in the ocelli (Wang et al. 2023) is a prime candidate for the regulation of light entrainment in response to the red light. The results for circadian entrainment in this study are in agreement with the identification of the photoreceptors that are involved in the circadian phase shift by light pulses (Wang et al. 2023). This suggests that both circadian entrainment by light–dark cycles and phase shift by light pulses are being regulated through the same photoreceptors.

Our results confirmed that *Nasonia* can entrain under red lights. We showed that all wasps were entrained under all three red LED lights (590, 625, 656 nm) when the T-cycles were close to their natural *tau* period (23 and 24 h). The percentage of entrainment steadily decreased when the T-cycles shortened. By putting wasps under extreme light dark cycle conditions (short T-cycles and light on–off schemes), we intended to challenge the circadian clock at the border of entrainment and free running. However, considering such unnatural light dark conditions, many wasps were in between partial entrainment and free running. We classified those animals without consistent entrainment pattern in their actograms and daily centre of gravity plots as free running to avoid overestimation of entrainment. Additionally, the lack of DD treatment limits the objectiveness in the classification of entrainment in our experiment. We focused on investigating the phase angle of entrainment instead to examine the strength of a *Zeitgeber*. The phase angle of entrainment under different T-cycles indicates the range of entrainment of a *Zeitgeber*, thus reflecting the strength of a *Zeitgeber*, as the range of entrainment widens with increasing *Zeitgeber* strength (Floessner and Hut 2017). Surprisingly, there was no significant difference in the phase angle and range of entrainment among the three red lights, suggesting the equivalence of the *Zeitgeber* strength of these three red LED lights. The wide emission spectrum of 590 nm LED light indicated that it can trigger the response of both green and ocellar red opsin photoreceptor cells. This may explain the similarity of the *Zeitgeber* strength of the three LED lights, such that both the green and ocellar red opsin classes mediated long-wavelength entrainment.

To compare the photopigments required for light entrainment by light–dark cycles and phase shift by light pulses and assess their relative sensitivities for light entrainment, we entrained *Nasonia* under nine different LED lights and five to six different light intensities of each wavelength. Consistent with our hypothesis, the non-univariance forms of the dose–response curves indicated that multiple photoreceptors are involved in the mediation of photoentrainment. The near or complete absence of a clear dose response at 528 nm and 505 nm may suggest that this range of light saturated the photoreceptors or the components of the downstream neural pathways at all light intensities, indicating the cumulative peak sensitivity of the circadian photoreceptors and the neurons that mediate the interactions between them. An alternative explanation for this could be that there is antagonistic interaction between different photoreceptors, which resulted in the absence of dose responses around 500–530 nm, similar to our previous results (Wang et al. 2023). The almost reversed pattern in the circadian action spectra between ED30 and ED80 also strengthens the possibility of such interactions between photoreceptors. The regulation of entrainment in the green range is independent of light intensity, as colour vision (which is defined as the ability of discrimination of light spectral content) is also independent of light intensity. Further experiments with lower light intensities are needed to distinguish between these alterative theories.

Unfortunately, our experimental setup did not allow us to narrow down the wavelengths of peak sensitivity for each of the photoreceptors. The commercially available LED lights were useful to explore the range of sensitivities, as they are easy to obtain and enabled us to test whether wasps can be light entrained under both narrow- and wide-ranging spectra. However, due to limited wavelengths available and the broader bandwidth of LED lights compared to monochromatic light sources, we cannot accurately pinpoint the peak sensitivities of each photoreceptor for light-induced entrainment. Additionally, to avoid temperature entrainment with our experimental setup, we applied a “10 min on—20 min off” type of cycle during the light phase of the LD cycles. Therefore, it is possible that the percentage of entrainment in *Nasonia* would be higher under normal LD cycles. Because of such potentially challenging LD cycles in our experiments, we intentionally prolonged the entrainment period to three weeks, to be able to induce stable entrainment. However, releasing animals back into constant darkness after LD cycles is also a crucial way of demonstrating entrainment. Considering the short life span of *Nasonia*, future experiments may need to shorten the period of LD cycles and follow this with a period of constant darkness, to unequivocally show entrainment.

The derived action spectra consistently demonstrated that the peak of cumulative sensitivity is within the blue and green range (~ 470–528 nm) of the spectrum which was independent of light intensity. This is similar to the cumulative peak sensitivity in the electroretinogram responses of the compound eyes. These results suggest that the blue- and green-sensitive photoreceptors are the main photoreceptors in the regulation of circadian entrainment by light dark cycles, similar to phase shift by light pulses.

Furthermore, our behavioural action spectra results revealed that the sensitivity to red light (> 590 nm) was strongly dependent on light intensity. Wasps were insensitive to the dim red light, but bright red light induced increasing percentages of entrainment, up to 100%. Based on electroretinogram measurements of the compound eye, the sensitivity to long-wavelength red light was low and independent of intensity. On the other hand, this result explains the dynamic changes observed in the electroretinogram recordings of the ocelli in the red range of the spectrum (Wang et al. 2023). This suggests that the photoreceptors for red light may be located elsewhere than in the compound eyes. It is likely that this receptor is located in the ocelli, since its spectral sensitivity in entrainment by light–dark cycles is also similar to that of circadian phase shift by light pulses. However, as it is possible for red light-insensitive photoreceptors to mediate circadian red-light entrainment, we cannot rule out the possibility of other photoreceptors such as the retinal green photoreceptor contributing to red-light entrainment. Indeed, it has been shown that other insects such as *Drosophila* can entrain to red light without having red light-sensitive photoreceptors (Senthilan et al. 2019). We can also not rule out that potential neuronal photoreceptors may exist and contribute to circadian entrainment in *Nasonia*. Nonetheless, together with previous research, our results confirmed that insects can perceive red light and use this for circadian entrainment and photoperiodism (Hanai et al. 2008, Lees 1981, Nelson et al. 2009, Saunders 1975).

Interestingly, our current results showed that *Nasonia*’s relative sensitivity for each wavelength changed under different levels of light intensity for entrainment, particularly in the red region of the spectrum. On the contrary, the overall sensitivity pattern across the spectrum for circadian phase shift by light pulses was largely independent of light intensity (Wang et al. 2023). This discrepancy might be explained by the fact that there are different mechanisms downstream of the same photoreceptors in the regulation of light entrainment by light–dark cycles and phase shift by light pulses. In *Drosophila*, multiple photoreceptors contribute to circadian entrainment, but detect different kinds of light information and convey the signals to the central oscillator through different mechanisms (Helfrich-Förster 2020). These mechanisms in *Drosophila* can be mediated through a deep brain photoreceptor (dCRY) that is extremely sensitive and can detect very dim light directly for circadian entrainment (Vinayak et al. 2013), but is insensitive to red light (Berndt et al. 2007; Hanai et al. 2008; van Vickle-Chavez and van Gelder 2007). In addition, several other light-sensitive molecules in *Drosophila* are shown to detect low light intensity and mediate re-entrainment (*Rhodopsin 1* [*Rh1*], *Rh3*, *Rh4*, *Rh5*, and *Rh6*) (Szular et al. 2012; Saint-Charles et al. 2016). *Rh1-* and *Rh6*-expressing photoreceptor cells are required for specific circadian red-light entrainment (Alejevski et al. 2019; Hanai et al. 2008). *Rh1*, *Rh5*, and *Rh6,* expressing photoreceptor cells, also mediates light resetting employing a novel non-canonical phototransduction pathway (Ogueta et al. 2018). It is possible that such a complex phototransduction mechanism also exists in *Nasonia* and that the regulation of circadian entrainment by light–dark cycles and phase shift by light pulses are regulated through different pathways.

Taken together, our results revealed that there is a general abundance of circadian photoreceptors and that insects can use a wide spectrum of light for daily circadian regulation. The mechanisms underlying light signal detection are dependent on light intensity and the two major photoreceptors (blue and green) are sensitive to a large range of light intensities. In contrast, the red light-sensitive photoreceptor requires higher light intensity for activation. We hypothesized that the ocellar red photoreceptor may enable *Nasonia* to use red light as a *Zeitgeber*. Our results showed that *Nasonia* can indeed be entrained under red light up until 656 nm, even under strenuous unnatural T-cycles that challenge entrainment of the endogenous clock. It is likely that the circadian system of *Nasonia* can be much more influenced by the prolonged (artificial) light exposure in the wild as well as in experimental conditions. This has immediate repercussion for researchers that handle insects under red light as a control condition or assuming it to constitute total darkness. Yet, we did not solve the question whether the sensory pathway underlying this red-light response relies on the ocellar red photoreceptor solely or that both green and ocellar red photoreceptors are involved. On the other hand, we can also not rule out that potential neuronal photoreceptors may be involved in the circadian photoreception. However, in *Nasonia* there is no homologue to *pteropsin* like in honeybees (Velarde et al. 2005), suggesting it lacks a neuronal photoreceptor. To further answer this question and investigate the different roles of photoreceptors in circadian photoentrainment, loss-of-function mutants of rhodopsin genes in combination with behavioural analysis under different light settings would provide more insights.

### Supplementary Information

Below is the link to the electronic supplementary material.

Supplementary file1 (ZIP 19 KB)

Supplementary file2 (PDF 2676 KB)

## Data Availability

All raw data was first processed in R as described in the methods and R script is included in the supplementary information. All processed data used in the further statistical analysis and figures are provided in the supplementary information. Raw behavioural data is available upon request.
