# Supplementary material for: Circadian entrainment to red-light Zeitgebers and action spectrum for entrainment in the jewel wasp Nasonia vitripennis
Source: J Comp Physiol A Neuroethol Sens Neural Behav Physiol. 2023 Sep 22;210(3):459–72. doi: 10.1007/s00359-023-01672-4 (PMC11106113; doi:10.1007/s00359-023-01672-4)
Supplement: Supplementary file 2 — Supplementary file2 (PDF 2676 KB) [file 359_2023_1672_MOESM2_ESM.pdf]

Circadian entrainment to red light *Zeitgebers* and action spectrum for entrainment in the jewel wasp *Nasonia vitripennis*

Journal of Comparative Physiology A

Yifan Wang[1*], Lijing Jin[1], Gregor Belušič[2], Leo W. Beukeboom[1], Bregje Wertheim[1*], Roelof A. Hut[1]

[1]Groningen Institute for Evolutionary Life Sciences, University of Groningen, 9712 CP Groningen, the Netherlands.

[2]Department of Biology, Biotechnical Faculty, University of Ljubljana, 1000 Ljubljana, Slovenia.

*Corresponding author email address: yifan.wang@rug.nl & b.wertheim@rug.nl

Supplements

Tab s1 Summary results of red-light entrainment experiments. For each treatment condition (i.e., combination of T cycle and LED light), the numbers of wasps that were scored as arrhythmic, entrained or free running is provided. Wasps that died within 10 days of the start of the experiment were scored as dead.

		arrhythmic		dead		entrained		free running	
wavelength	Tcycle	count	percentage	count	percentage	count	percentage	count	percentage
590	24	2	6%	3	9%	27	84%	0	0%
	23	3	9%	10	31%	19	59%	0	0%
	22	3	9%	2	6%	24	75%	3	9%
	21	4	13%	11	34%	10	31%	7	22%
	20	6	19%	12	38%	7	22%	7	22%
	19	7	22%	7	22%	6	19%	12	38%
625	24	0	0%	9	28%	23	72%	0	0%
	23	1	3%	14	44%	17	53%	0	0%
	22	2	6%	11	34%	15	47%	4	13%
	21	4	13%	11	34%	11	34%	6	19%
	20	3	9%	11	34%	1	3%	17	53%
	19	3	9%	12	38%	0	0%	17	53%
656	24	1	3%	12	38%	19	59%	0	0%
	23	3	9%	17	53%	12	38%	0	0%
	22	2	6%	9	28%	17	53%	4	13%
	21	4	13%	7	22%	14	44%	7	22%
	20	4	13%	10	31%	1	3%	17	53%
	19	9	28%	8	25%	1	3%	14	44%

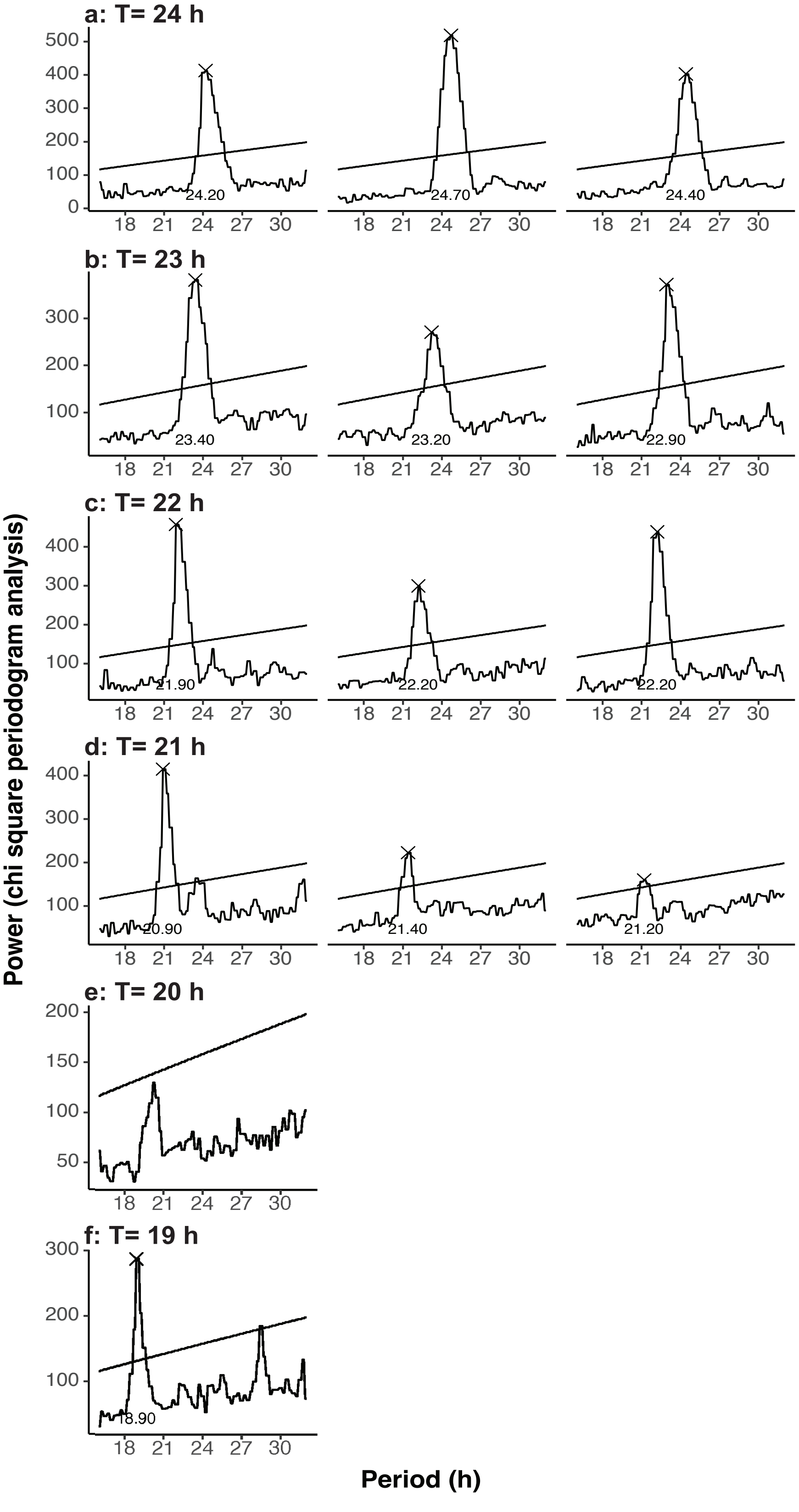

Fig s1 Representative periodograms of entrained *Nasonia* under different T-cycles under far-red light (656 nm). a-f Three representative periodograms of the same entrained wasps as in Fig. 1 under each T-cycle ranging from a 24 T to f 19 T. Within each graph, the statistical power from the Chi-square

periodogram analysis is plotted against the period (h). The period for each animal is calculated with R package Zeitgeber and is labelled inside each graph.

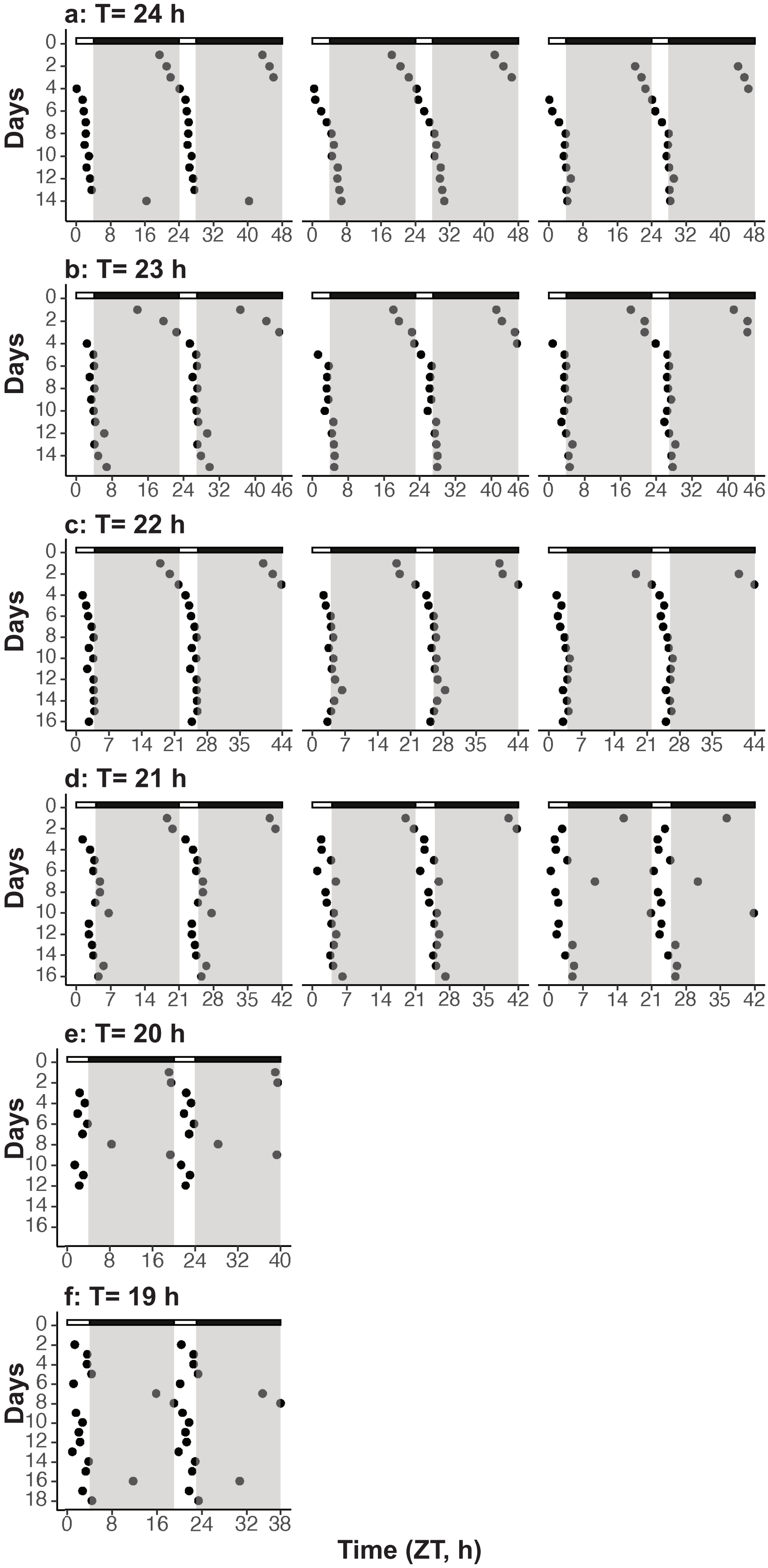

a: T= 24 h
b: T= 23 h
c: T= 22 h
d: T= 21 h
e: T= 20 h
f: T= 19 h
Days
Time (ZT, h)

Fig s2 Representative daily centre of gravity plots of entrained *Nasonia* under different T-cycles under far-red light (656 nm). a-f Three representative centre of gravity in double-plot of the same entrained wasps as in Fig. 1 under each T-cycle ranging from a 24 T to f 19 T. T-cycle length is the sum of the light and dark phase lengths. The light phase was kept constant at 4 h and only the dark phase varied in different T-cycle experiments. Light/dark cycle is indicated on top and in the background of each panel where white is the light phase and black/grey is the dark phase. Time scale starts at ZT0, the start of light on. Within each graph, each dot indicates the centre of gravity of the activity for each day under the LD cycle.

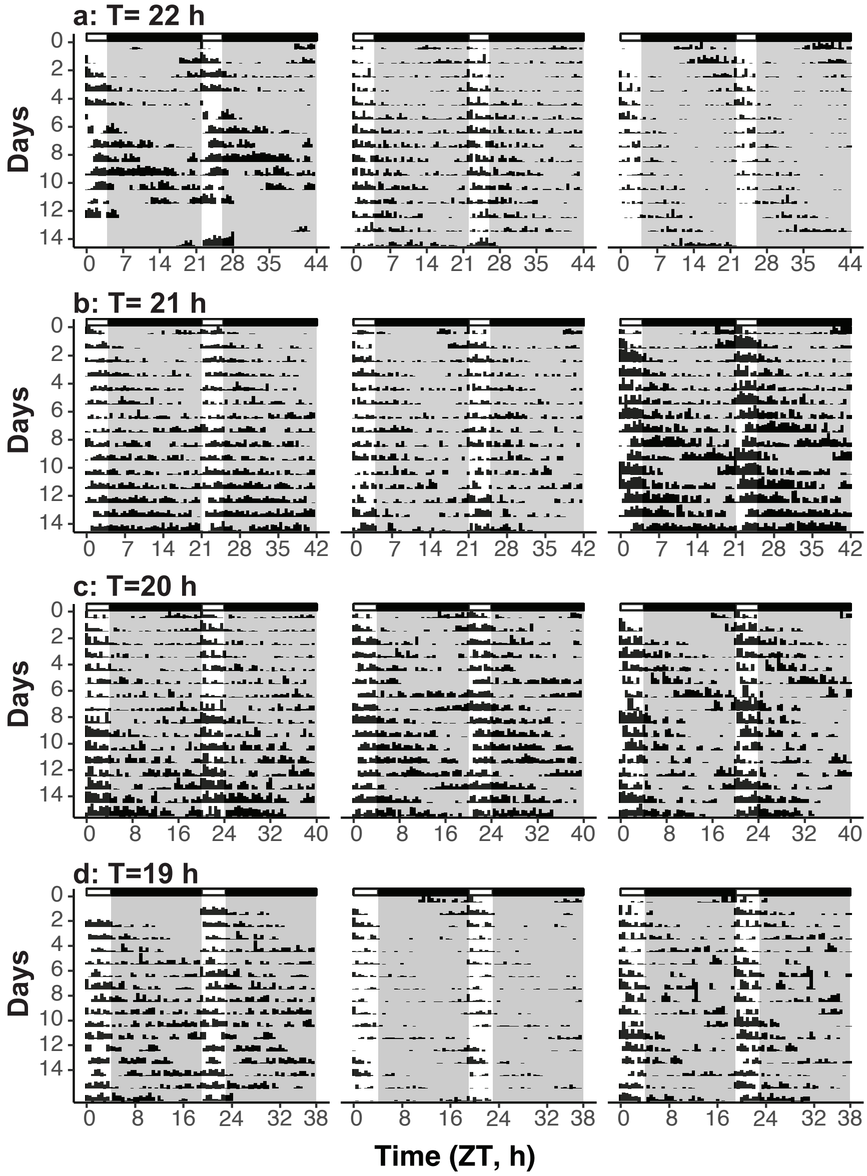

Fig s3 Representative activity patterns of free running *Nasonia* under different T-cycles under far-red light (656 nm). a-d Three representative actograms in double-plot of *Nasonia* free running under each T-cycle ranging from a 22 T to d 19 T. T-cycle length is the sum of the light and dark phase lengths. The light phase was kept constant at 4 h and only the dark phase varied in different T-cycle experiments. Light/dark cycle is indicated on top and in the background of each panel where white is the light phase and black/grey is the dark phase. Time scale starts at ZT0, the start of light on.

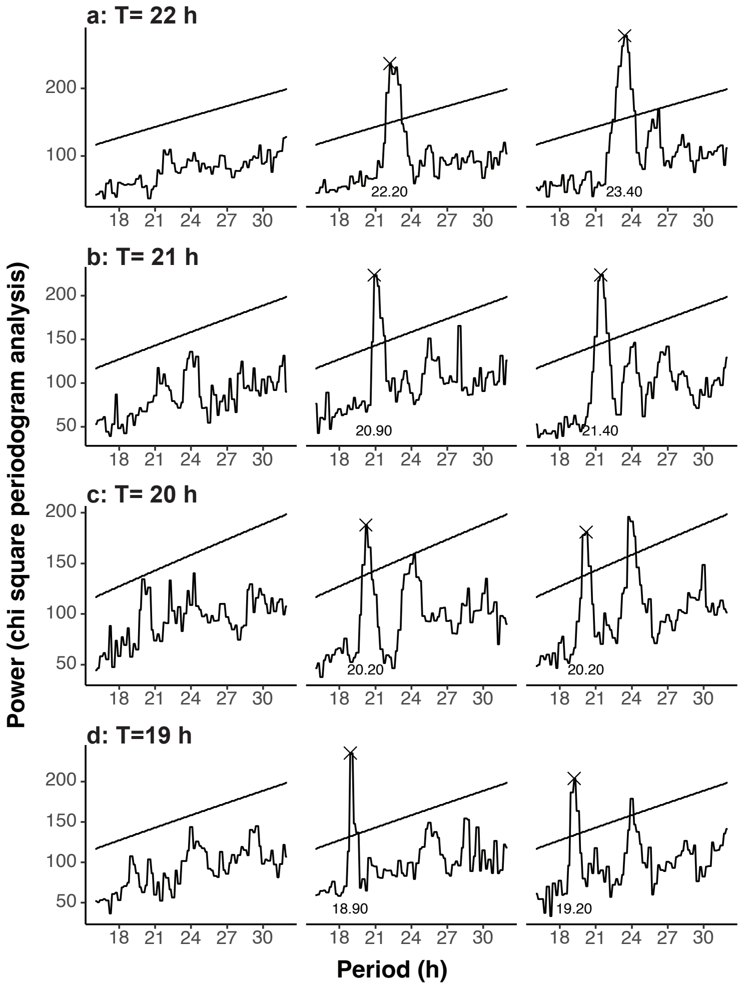

Fig s4 Representative periodograms of free running *Nasonia* under different T-cycles under far-red light (656 nm). a-d Three representative periodograms of the same free running wasps as in Fig. s3 under each T-cycle ranging from a 22 T to d 19 T. Within each graph, the statistical power from the Chi-square periodogram analysis is plotted against the period (h). The period for each animal is calculated with R package Zeitgeber and is labelled inside each graph.

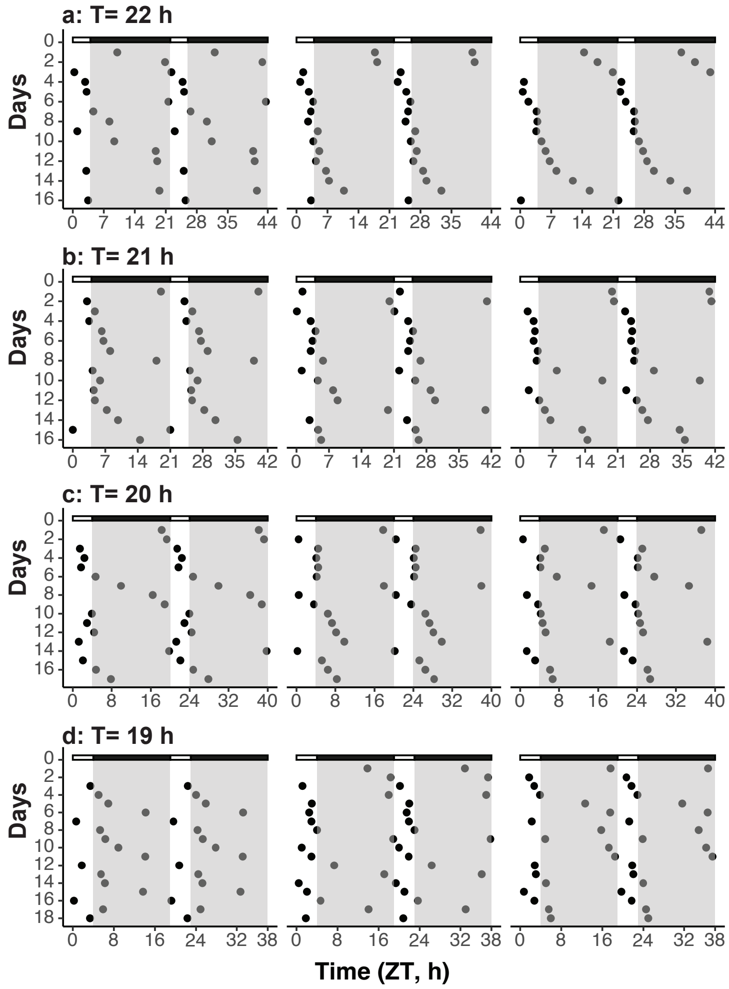

Fig s5 Representative daily centre of gravity plots of free running *Nasonia* under different T-cycles under far-red light (656 nm). a-d Three representative centre of gravity in double-plot of the same free running wasps as in Fig. s3 under each T-cycle ranging from a 22 T to d 19 T. T-cycle length is the sum of the light and dark phase lengths. The light phase was kept constant at 4 h and only the dark phase varied in different T-cycle experiments. Light/dark cycle is indicated on top and in the background of each panel where white is the light phase and black/grey is the dark phase. Time scale starts at ZT0, the start of light on. Within each graph, each dot indicates the centre of gravity of the activity for each day under the LD cycle.

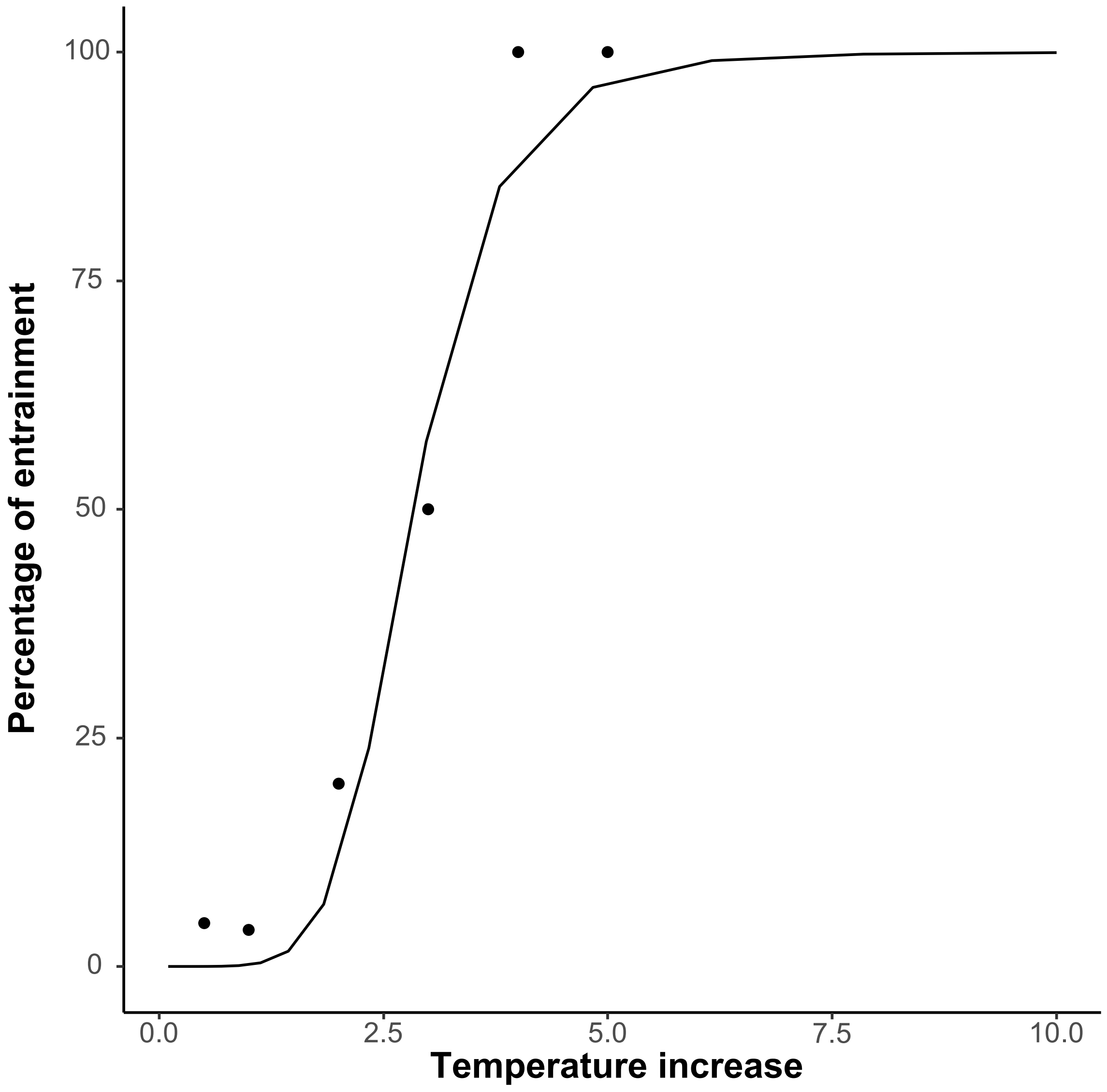

Fig s6 Temperature entrainment of *Nasonia* under six different temperature cycles ranging from 0.5 °C to 5 °C increase. *Nasonia* was entrained under a cycle of 20 hours of constant temperature (18 °C) and 4 hours of raised temperature (18.5 °C to 23 °C). The percentage of entrainment of *Nasonia* under all temperature conditions is calculated as the number of entrained *Nasonia* divided by the total number of rhythmic animals (entrained and free running).

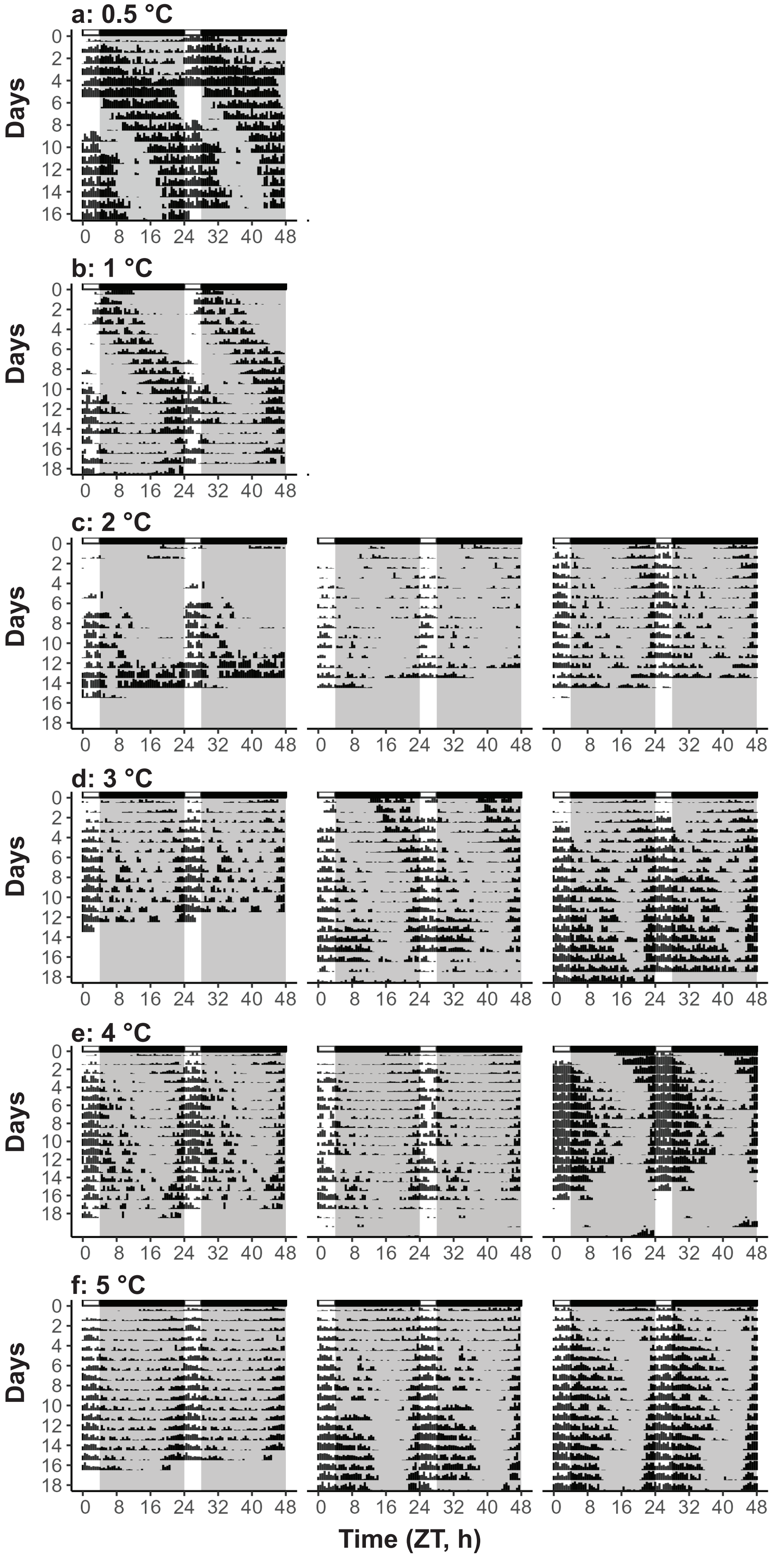

a: 0.5 °C
b: 1 °C
c: 2 °C
d: 3 °C
e: 4 °C
f: 5 °C
Days
0
2
4
6
8
10
12
14
16
18
0 8 16 24 32 40 48
Time (ZT, h)

Fig s7 Representative activity patterns of entrained *Nasonia* under different temperature cycles ranging from 0.5 °C to 5 °C increase. a-f Three representative actograms in double-plot of *Nasonia* entrained under different temperature cycles. Time scale starts at ZT0 as temperature increases. The duration of the warm phase was 4 h and the duration of the cold phase was 20 h. The cold phase was at a constant 18 ˚C, whereas the warm phase was an increase of 0.5 ˚C to 5 ˚C on top of the constant temperature each time. Warm/cold cycle is indicated on top and in the background of each panel where white is the warm phase and black/grey is the dark phase. The entire experiment was conducted under total darkness.

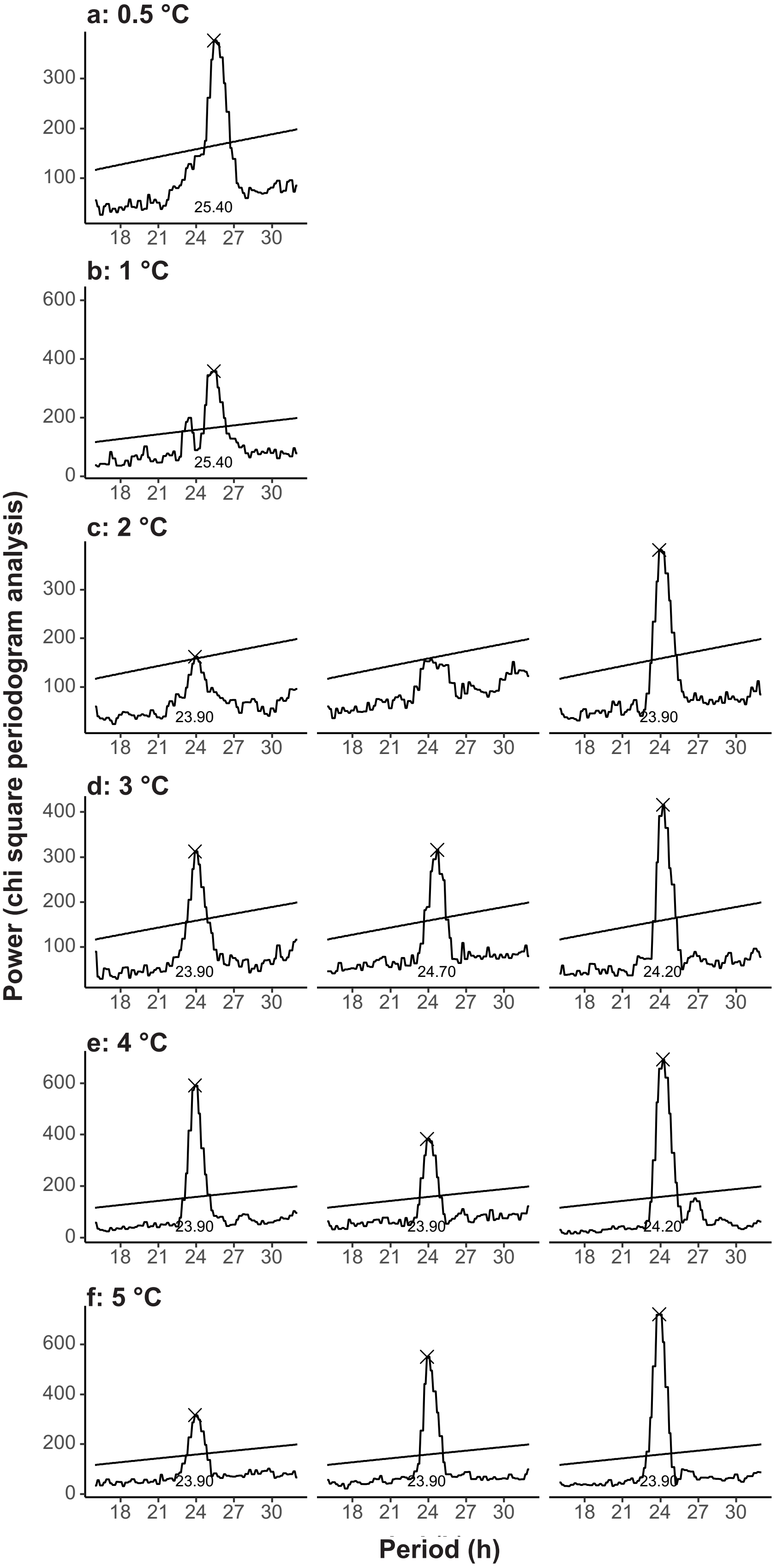

Fig s8 Representative periodograms of entrained *Nasonia* under different temperature cycles ranging from 0.5 °C to 5 °C increase. a-f Three representative periodograms of the same entrained wasps as

in Fig. s7 under each temperature cycle ranging from a 0.5 °C to f 5 °C increase. Within each graph, the statistical power from the Chi-square periodogram analysis is plotted against the period (h). The period for each animal is calculated with the R package Zeitgeber and is labelled inside each graph.

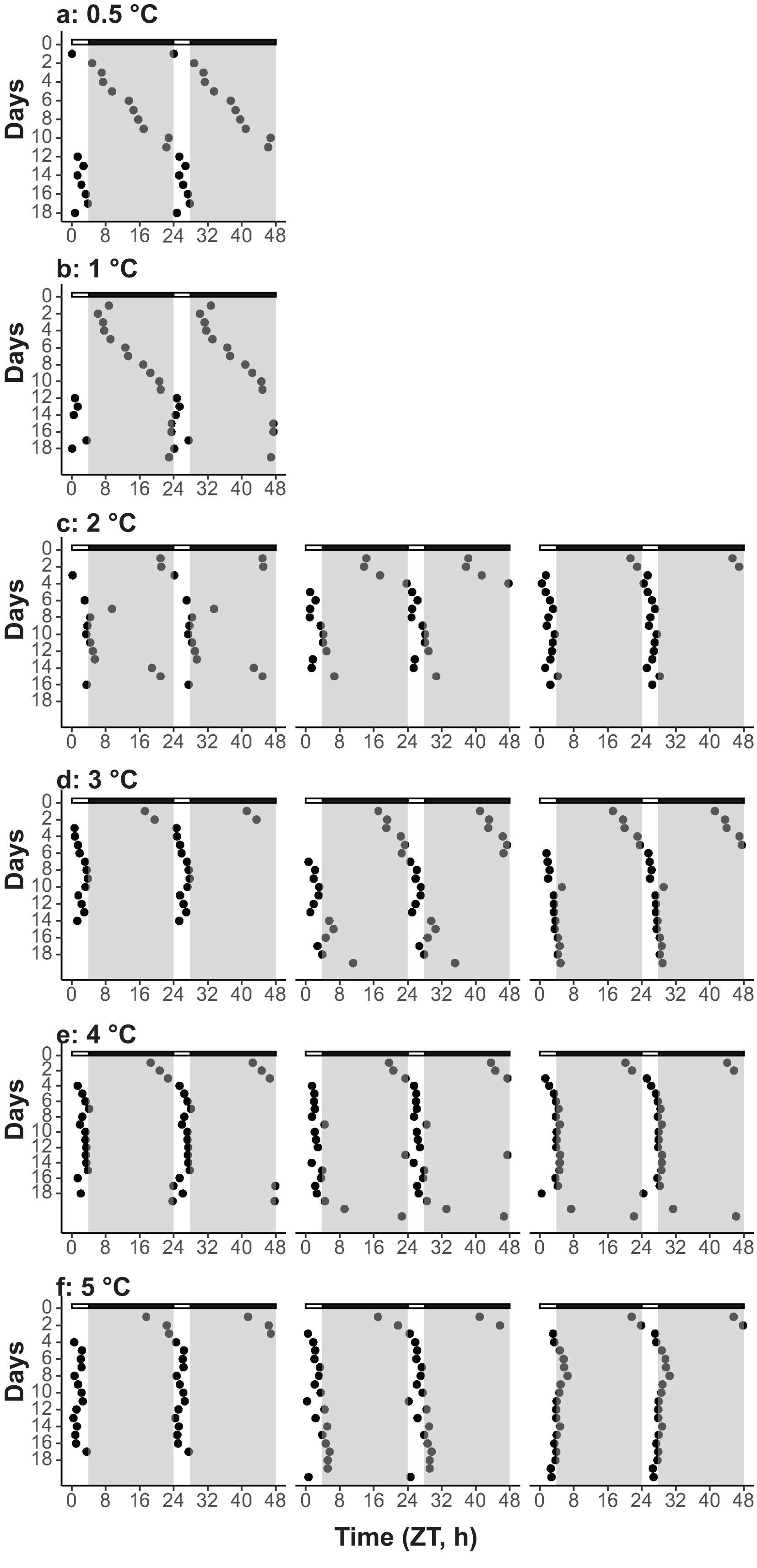

Fig s9 Representative daily centre of gravity plots of entrained *Nasonia* under different temperature cycles ranging from 0.5 °C to 5 °C increase**.** a-f Three representative centre of gravity in double-plot of

the same entrained wasps as in Fig. s7 under each temperature cycle ranging from a 0.5 °C to f 5 °C increase. The duration of the warm phase was 4 h and the duration of the cold phase was 20 h. The cold phase was at a constant 18 ˚C, whereas the warm phase was an increase of 0.5 ˚C to 5 ˚C on top of the constant temperature each time. Warm/cold cycle is indicated on top and in the background of each panel where white is the warm phase and black/grey is the dark phase. Time scale starts at ZT0, the start of temperature increase. Within each graph, each dot indicates the centre of gravity of the activity for each day under the LD cycle.

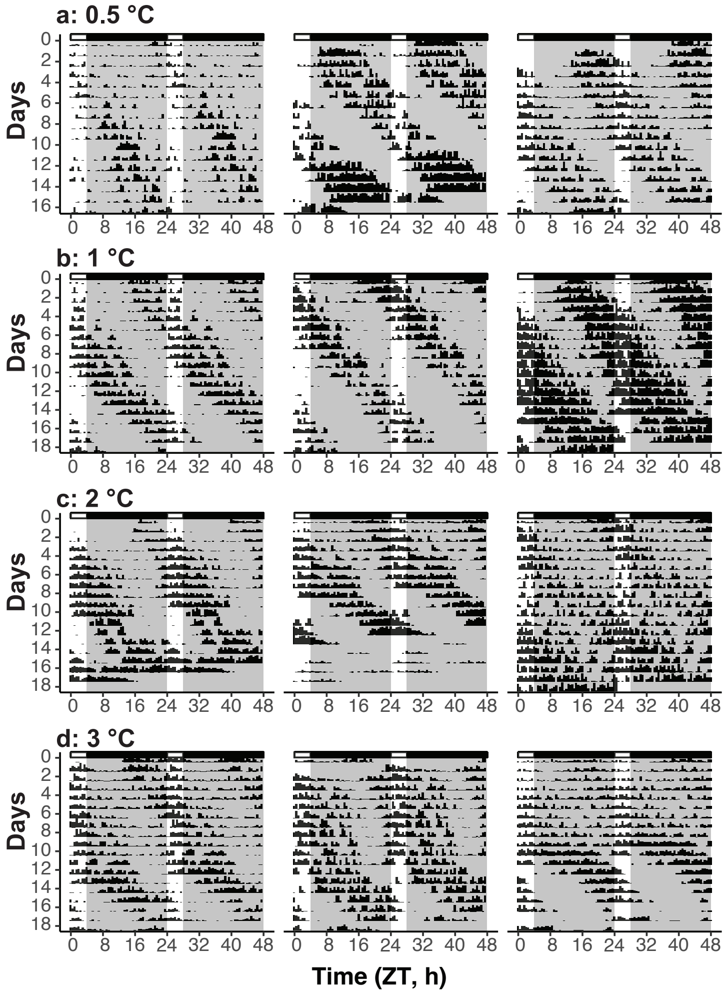

Fig s10 Representative activity patterns of free running *Nasonia* under different temperature cycles ranging from 0.5 °C to 3 °C increase. a-d Three representative actograms in double-plot of *Nasonia* free running under different temperature cycles. Time scale starts at ZT0 as temperature increases on. The duration of the warm phase was 4 h and the duration of the cold phase was 20 h. The cold phase was at a constant 18 ˚C, whereas the warm phase was an increase of 0.5 ˚C to 3 ˚C on top of the constant temperature each time. Warm/cold cycle is indicated on top and in the background of each panel where white is the warm phase and black/grey is the dark phase. The entire experiment was conducted under total darkness.

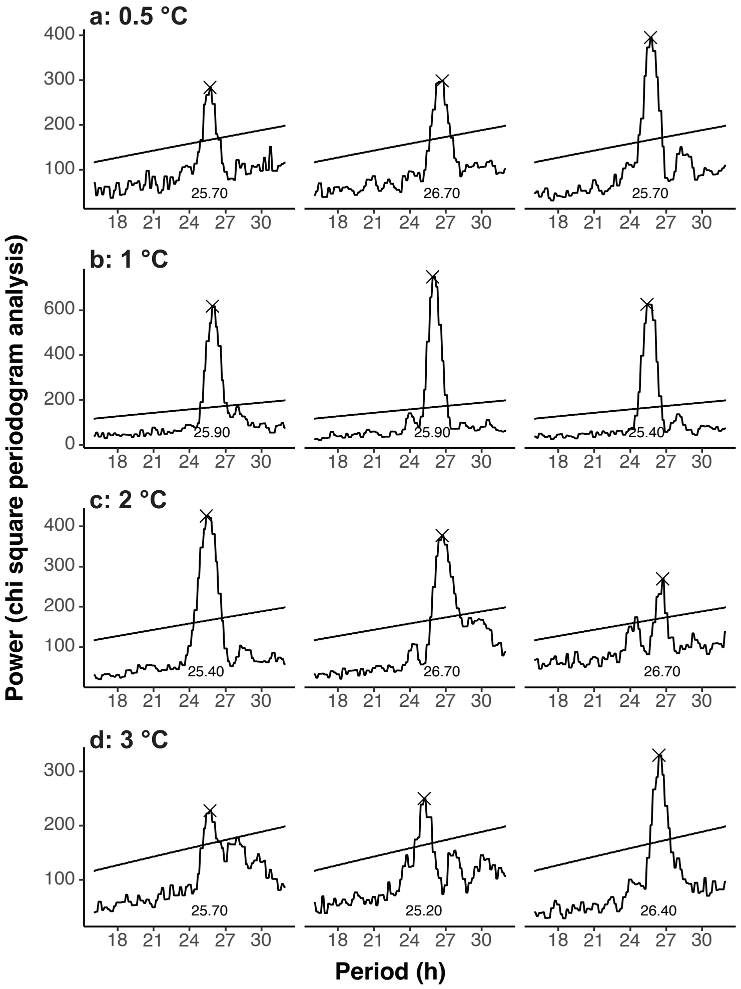

Fig s11 Representative periodograms of free running *Nasonia* under different temperature cycles ranging from 0.5 °C to 3 °C increase**.** a-d Three representative periodograms of the same free running wasps as in Fig. s10 under each temperature cycle ranging from a 0.5 °C to d 3 °C increase. Within each graph, the statistical power from the Chi-square periodogram analysis is plotted against the period (h). The period for each animal is calculated with the R package Zeitgeber and is labelled inside each graph.

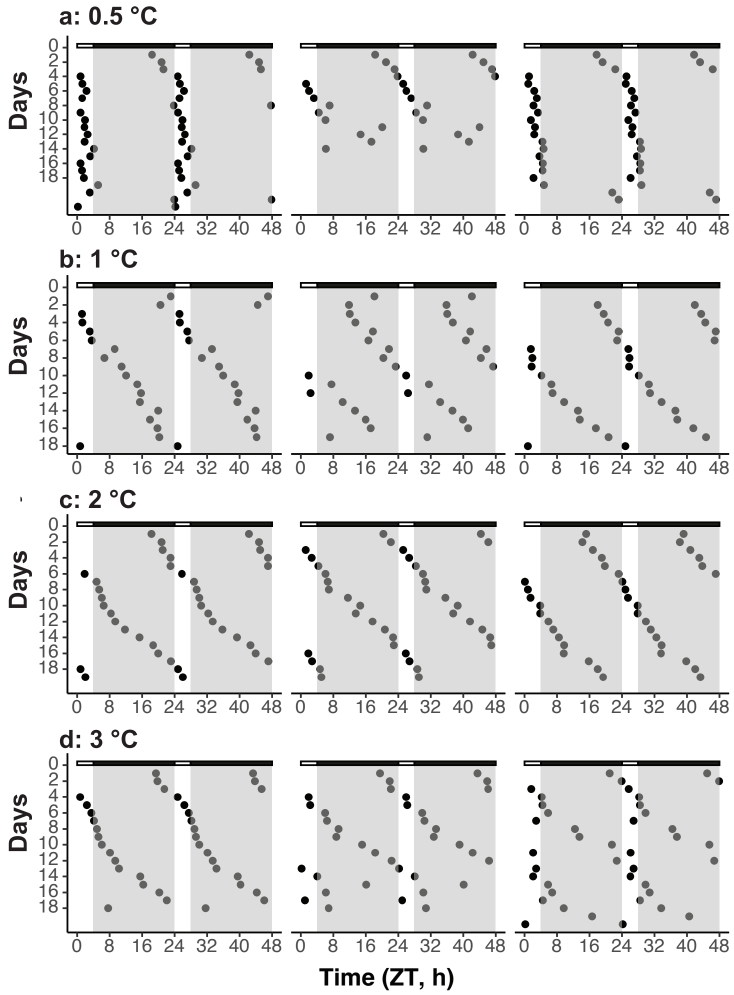

Fig s12 Representative daily centre of gravity plots of free running *Nasonia* under different temperature cycles ranging from 0.5 °C to 3 °C increase. a-d Three representative centre of gravity in double-plot of the same free running wasps as in Fig. s10 under each temperature cycle ranging from a 0.5 °C to d 3 °C increase. The duration of the warm phase was 4 h and the duration of the cold phase was 20 h. The cold phase was at a constant 18 ˚C, whereas the warm phase was an increase of 0.5 ˚C to 3 ˚C on top of the constant temperature each time. Warm/cold cycle is indicated on top and in the background of each panel where white is the warm phase and black/grey is the dark phase. Time scale starts at ZT0, the start of temperature increase. Within each graph, each dot indicates the centre of gravity of the activity for each day under the LD cycle.

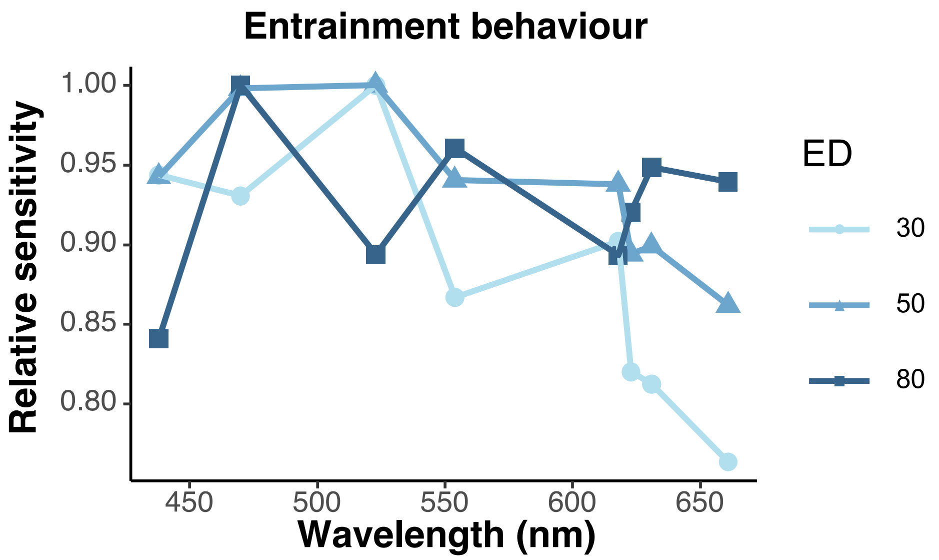

Fig s13 Circadian action spectra of entrainment behaviour (with more precise peak wavelengths). This action spectra of entrainment behaviour were derived from the dose-response curves in Fig. 3a by determining the light intensities that were necessary to entrain 30%, 50%, and 80% of the wasps at each wavelength. The effective dose of 505 nm was excluded from action spectra as the dose-response curve was unable to fit in that data. The peak wavelengths measured with a spectrometer were plotted in the x-axis in this figure instead of the manufacturer reported wavelengths. Relative sensitivity is calculated as 1/effective dose (light intensity) and normalized to the maximum sensitivity. The colours indicate ED30 (light blue), ED50 (blue line), and ED80 (dark blue line).

Tab s2 Summary results of temperature entrainment experiments. For each treatment condition (i.e., each temperature cycle), the numbers of wasps that were scored as arrhythmic, entrained or free running is provided. Wasps that died within 10 days of the start of the experiment were scored as dead.

	arrhythmic		dead		entrained		free running	
**temperature**	count	percentage	count	percentage	count	percentage	count	percentage
**0.5**	3	9%	8	25%	1	3%	20	63%
**1**	0	0%	7	22%	1	3%	24	75%
**2**	4	13%	13	41%	3	9%	12	38%
**3**	4	13%	14	44%	7	22%	7	22%
**4**	2	6%	6	19%	24	75%	0	0%
**5**	5	16%	8	25%	19	59%	0	0%

Tab s3 Summary results of different LED lights entrainment experiments. For each treatment condition (i.e., combination of LED lights and different light intensities), the numbers of wasps that were scored as arrhythmic, entrained or free running is provided. Wasps that died within 10 days of the start of the experiment were scored as dead.

		arrhythmic		dead		entrained		free running	
wavelength	light intensity	count	percentage	count	percentage	count	percentage	count	percentage
455	1.41E+13	8	25%	8	25%	8	25%	8	25%
	2.58E+13	0	0%	5	16%	11	34%	16	50%
	5.31E+13	11	17%	15	23%	21	33%	17	27%
	2.52E+14	3	9%	8	25%	11	34%	10	31%
	2.47^{E}+15	4	13%	6	19%	15	47%	7	22%
470	7.94E+13	5	16%	4	13%	9	28%	14	44%
	2.09E+13	3	9%	4	13%	15	47%	10	31%
	3.30E+13	2	6%	10	31%	14	44%	6	19%
	1.85E+14	1	3%	7	22%	20	63%	4	13%
	2.36E+15	11	17%	9	14%	31	48%	13	20%
505	6.25E+12	8	25%	11	34%	4	13%	9	28%
	1.50E+13	4	13%	2	6%	16	50%	10	31%
	2.85E+13	8	13%	17	27%	19	30%	20	31%
	1.25E+14	5	16%	9	28%	11	34%	7	22%
	1.78E+15	6	19%	3	9%	9	28%	14	44%
528	4.89E+12	3	9%	12	38%	6	19%	11	34%
	1.91E+13	4	13%	7	22%	15	47%	6	19%
	3.84E+13	11	17%	22	34%	17	27%	14	22%
	1.79E+14	2	6%	5	16%	17	53%	8	25%
	1.67E+15	4	13%	6	19%	14	44%	8	25%
566	2.19E+13	8	25%	9	28%	4	13%	11	34%
	3.76E+13	3	9%	5	16%	15	47%	9	28%
	4.62E+13	5	16%	7	22%	11	34%	9	28%
	1.53E+15	10	16%	28	44%	15	23%	11	17%
	1.86E+15	3	9%	5	16%	22	69%	2	6%
590	1.83E+13	4	13%	12	38%	4	13%	12	38%
	3.20E+13	8	25%	8	25%	8	25%	8	25%
	5.10E+13	6	19%	8	25%	13	41%	5	16%
	1.75E+15	9	14%	18	28%	18	28%	19	30%
	2.17E+15	2	6%	3	9%	22	69%	5	16%
617	1.63E+13	11	34%	4	13%	2	6%	15	47%
	3.27E+13	1	3%	1	3%	8	25%	22	69%
	5.20E+13	5	16%	12	38%	7	22%	8	25%
	2.78E+14	2	6%	5	16%	18	56%	7	22%
	2.75E+15	13	20%	12	19%	21	33%	18	56%
625	1.16E+13	10	31%	3	9%	2	6%	17	53%
	2.11E+13	0	0%	3	9%	7	22%	22	69%
	4.40E+13	12	19%	20	31%	10	16%	22	34%
	2.11E+14	2	6%	6	19%	17	53%	7	22%
	2.22E+15	4	13%	11	34%	11	34%	6	19%
	2.62E+15	5	16%	10	31%	12	38%	5	16%
656	2.37E+13	7	22%	10	31%	0	0%	15	47%
	6.41E+13	8	25%	5	16%	0	0%	15	47%
	1.45E+14	10	16%	19	30%	15	23%	20	31%
	5.50E+14	2	6%	10	31%	8	25%	12	38%
	3.30E+15	4	13%	7	22%	14	44%	7	22%
	4.04E+15	4	13%	7	22%	16	50%	5	16%

Tab s4 Dose-response analysis model estimation.

Parameter	Estimate	Std. Error	t-value	p- value
**Slope:455 nm**	-5.32451	5.07969	-1.0482	0.304194
**Slope:470 nm**	-9.62326	7.98904	-1.2046	0.239225
**Slope:528 nm**	-5.36035	5.01934	-1.0679	0.295363
**Slope:566 nm**	-11.09259	5.84586	-1.8975	0.068918 .
**Slope:590 nm**	-7.11458	4.80703	-1.4800	0.150880
**Slope:617 nm**	-11.84930	6.12383	-1.9349	0.063946 .
**Slope:625 nm**	-14.96294	5.24752	-2.8514	0.008415 **
**Slope:656 nm**	-23.10590	7.19262	-3.2124	0.003493 **
**ED50:455 nm**	13.64124	0.77171	17.6766	4.829e-16 ***
**ED50:470 nm**	12.88306	0.64185	20.0719	< 2.2e-16 ***
**ED50:528 nm**	12.85692	0.97885	13.1347	5.516e-13 ***
**ED50:566 nm**	13.67231	0.39682	34.4545	< 2.2e-16 ***
**ED50:590 nm**	13.71105	0.59554	23.0228	< 2.2e-16 ***
**ED50:617 nm**	14.38111	0.43380	33.1516	< 2.2e-16 ***
**ED50:625 nm**	14.30177	0.30475	46.9294	< 2.2e-16 ***
**ED50:656 nm**	14.91959	0.22872	65.2305	< 2.2e-16 ***
